# EVALUATING THE ENGAGING OLDER ADULT LEARNERS AS HEALTH RESEARCHERS PROJECT I

**DOI:** 10.1093/geroni/igz038.1503

**Published:** 2019-11-08

**Authors:** Kate Kondolf, Joyce Duckles, sandhya Seshadri, Phyllis Jackson, Craig Sellers, Doreen Young, George Moses, Silvia Sörensen

**Affiliations:** 1 Warner School for Education and Human Development, University of Rochester, Rochester, New York, United States; 2 Warner School for Education and Human Development, Rochester, New York, United States; 3 University of Rochester School of nursing, Rochester, New York, United States; 4 Common Ground Health, Rochester, New York, United States; 5 Beechwood Neighborhood Center, Rochester, New York, United States; 6 North East Development Corporation, Rochester, New York, United States

## Abstract

We discuss the evaluation of the Engaging Older Adult Learners as Health Researchers (ENGOAL), a program designed to educate older adults from underserved and underresourced communities about geriatric health and research methods, enabling them to become Research Partners. Quantitative and qualitative data were collected for evaluation from 21 participants aged 53-79. We used All Aspects of Health Literacy Scale (AAHLS, King’s College, London) and an adaptation of Stanford Patient Education Research Center Chronic Disease Self-Efficacy Scale to assess pre-post and follow-up changes, using repeated measures analyses. Results suggest notable increases in self-efficacy, but only small improvements in subjective health literacy. Themes identified through qualitative analysis of interviews with participants included: (1) Finding our Voices (2) Race and Health (3) Faith and Health (4) Communicating with Providers and (5) Sharing and Advocacy. Emerging community leadership of participants are further evidence of confidence gains in our participants.

